# Microevolution of CG23-I Hypervirulent Klebsiella pneumoniae during Recurrent Infections in a Single Patient

**DOI:** 10.1128/spectrum.02077-22

**Published:** 2022-09-21

**Authors:** Yao-Chen Wang, Min-Chi Lu, Yia-Ting Li, Hui-Ling Tang, Pei-Yi Hsiao, Bo-Han Chen, Ru-Hsiou Teng, Chien-Shun Chiou, Yi-Chyi Lai

**Affiliations:** a Department of Internal Medicine, Chung Shan Medical University Hospital, Taichung, Taiwan; b School of Medicine, Chung Shan Medical University, Taichung, Taiwan; c Department of Microbiology and Immunology, School of Medicine, China Medical University grid.411508.9grid.254145.3 , Taichung, Taiwan; d Division of Infectious Diseases, Department of Internal Medicine, China Medical University Hospital, Taichung, Taiwan; e Division of Respiratory Therapy, Department of Internal Medicine, Chung Shan Medical University Hospital, Taichung, Taiwan; f Department of Microbiology and Immunology, School of Medicine, Chung Shan Medical University, Taichung, Taiwan; g Central Region Laboratory, Center for Diagnostics and Vaccine Development, Centers for Disease Control, Ministry of Health and Welfare, Taipei, Taiwan; National University Hospital

**Keywords:** *Klebsiella pneumoniae*, CG23-I, ST23, KL1, hypervirulent, *gyrA* mutation

## Abstract

CG23-I lineage constitutes the majority of hypervirulent Klebsiella pneumoniae. A diabetic patient suffered six episodes of infections caused by CG23-I K. pneumoniae. A total of nine isolates were collected in 2020. We performed whole-genome sequencing to elucidate the within-patient evolution of CG23-I K. pneumoniae. The maximum pairwise difference among the nine longitudinally collected isolates was five single nucleotide polymorphisms. One of the mutations was at the Asp87 position of GyrA. Four indels were identified, including an initiator tRNAfMet duplication, a tRNAArg deletion, a 7-bp insertion, and a 22-bp deletion. All 9 isolates had the genomic features of CG23-I K. pneumoniae, a chromosome-borne ICE*Kp10*, and a large virulence plasmid. The carriage of a complete set of genes for the biosynthesis of colibactin by ICE*Kp10* gave the nine isolates an ability to cause DNA damage to RAW264.7 cells. Compared with the initial isolate, the last isolate with an additional copy of initiator tRNA^fMet^ grew faster in a nutrient-limiting condition and exhibited enhanced virulence in BALB/c mice. Collectively, we characterized the within-patient microevolution of CG23-I K. pneumoniae through an in-depth comparison of genome sequences. Using the *in vitro* experiments and mouse models, we also demonstrated that these genomic alterations endowed the isolates with advantages to pass through *in vivo* selection.

**IMPORTANCE** CG23-I is a significant lineage of hypervirulent Klebsiella pneumoniae. This study characterizes the within-patient microevolution of CG23-I K. pneumoniae. Selective pressures from continuous use of antibiotics favored point mutations contributing to bacterial resistance to antibiotics. The duplication of an initiator tRNA^fMet^ gene helped CG23-I K. pneumoniae proliferate to reach a maximal population size during infections. For longer persistence inside a human host, the large virulence plasmid evolved with more flexible control of replication through duplication of the iteron-1 region. With the genomic alterations, the last isolate had a growth advantage over the initial isolate and exhibited enhanced virulence in BALB/c mice. This study gives us a deeper understanding of the genome evolution during the within-patient pathoadaptation of CG23-I K. pneumoniae.

## INTRODUCTION

Klebsiella pneumoniae is undoubtedly one of this century's toughest pathogens. With superior adaptability, K. pneumoniae survives in extreme environments and colonizes the mucosal tracts of humans and animals. The K. pneumoniae population is highly heterogeneous, consisting of more than 5,000 sequence types (STs). Some STs have become high-risk clones with drug resistance or hypervirulence development (1). The emergence of “hypervirulent” K. pneumoniae (HvKP) has been noticed since mid-1980 (2, 3). Unlike classic K. pneumoniae, HvKP frequently infects healthy individuals at any age, causes pyogenic liver abscesses, and develops metastatic complications, such as endophthalmitis, meningitis, pneumonia, necrotizing fasciitis, bacteremia, and septicemia (4). Immunocompromised individuals are highly susceptible to HvKP infections. Diabetes is a common risk factor for the development of metastatic complications (5) and is associated with relapse of HvKP infection that occurred months after initial therapy (6, 7). Although HvKP infections were initially prevalent in Southeast Asia, new cases have been increasingly identified in countries of other areas (8–11). Our knowledge regarding which combinations of genes are required for the maximal virulence of HvKP is still restricted. However, recent advances in the whole-genome sequencing (WGS) technique have allowed us to identify HvKP-specific genomic features and reveal the phylogenomic relatedness. Among the diverse STs identified so far, clonal group 23 (CG23), consisting of ST23, ST26, ST57, and ST163, constitutes 37% to 64% of HvKP in Asia (12).

Lam et al. have established a phylogenetic clustering scheme for CG23 HvKP (12). Over 80% of HvKP belonged to the sublineage I of CG23. CG23-I HvKP were clonally expanded with limited variation in the core genome and high conservation of the acquired virulence-associated genes. Besides displaying the serum-resistant K1-type CPS (13, 14), CG23-I HvKP harbors a pLVPK-like virulence plasmid (15, 16) and a chromosome-borne integrative conjugative element (ICE*Kp10*) that mobilizes the *ybt-1* (coding for biosynthesis of the siderophore yersiniabactin), and *clb-2* (coding for the genotoxin colibactin) loci (17). The production of colibactin is a virulence feature exclusively presented by CG23-I HvKP. Not only a genotoxin capable of inducing DNA double-strand breaks, cross-links, and chromosome instability, colibactin was also a virulence determinant for pathogenic Escherichia coli IHE3034 (18–20) and contributed to the development of meningitis caused by CG23-I HvKP in BALB/c mice (21, 22).

In 2020, a diabetic patient suffered recurrent infections caused by CG23-I HvKP with the clinical manifestations, from necrotizing fasciitis to psoas muscle abscess with infrarenal abdominal aortic aneurysm, and finally bacteremia-related multiorgan abscesses. Even with aggressive treatments, we could not eradicate the HvKP with a hidden niche to evolve and elicit various focal sites of infections in this patient. Although CG23-I K. pneumoniae has been known for its hypervirulence, we know little about how HvKP adapts inside human patients to counteract clinical treatments. To elucidate the within-patient microevolution of CG23-I HvKP, we collected nine isolates from this patient and determined the WGS of these isolates. Through in-depth comparison of genome sequences, *in vitro* experiments, and mouse infection models, we demonstrated that the isolates have evolved with genomic alterations. These alterations optimized their growth and hypervirulence and, ultimately, enabled them to pass through the *in vivo* selection within the patient.

## RESULTS

### Clinical manifestations.

**FIG 1:**
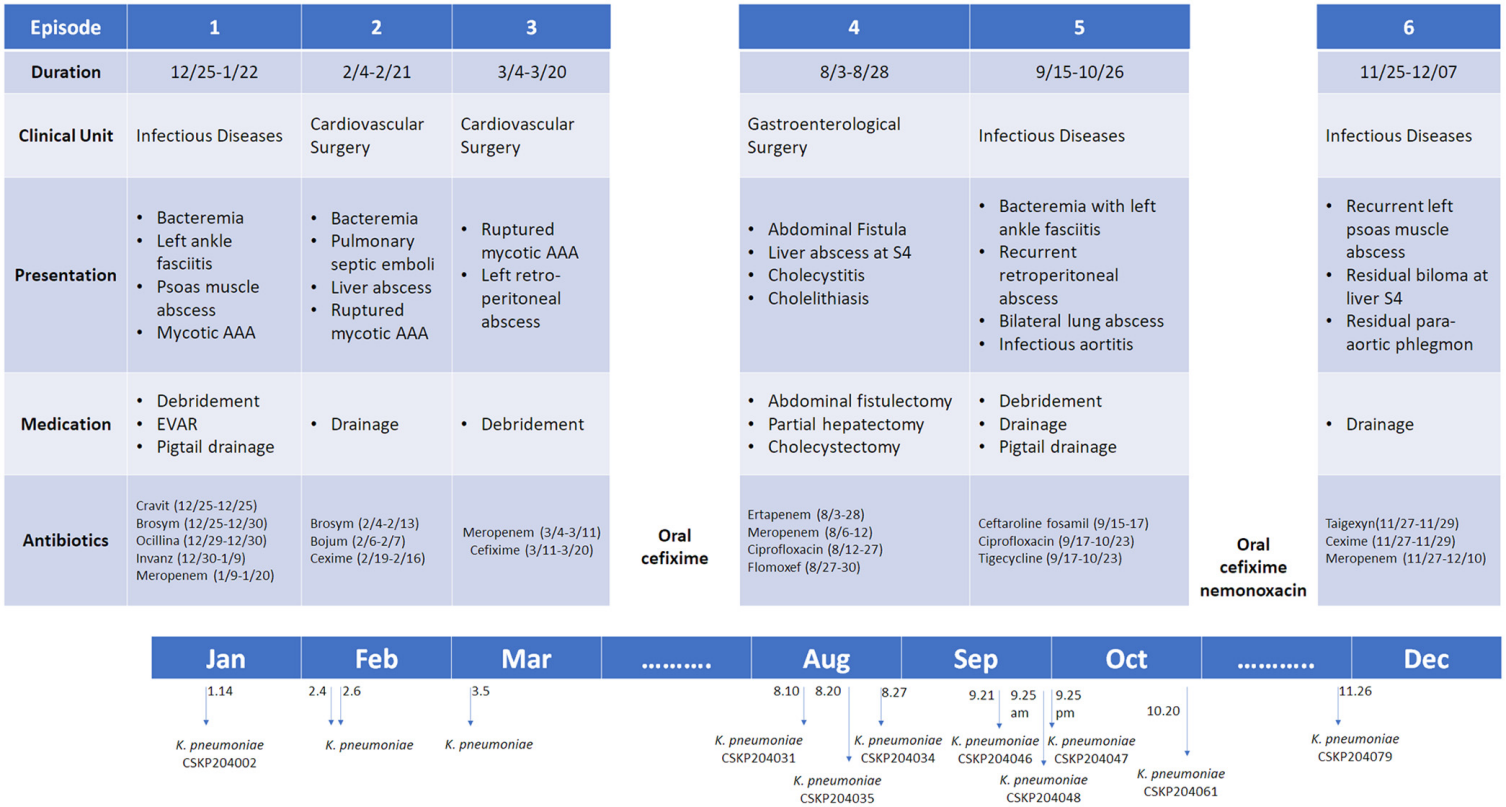
Timeline of the six episodes of K. pneumoniae infections in the patient, from December 2019 through December 2020. A total of nine isolates collected from the infectious foci were analyzed in this study. AAA, abdominal aortic aneurysm; EVAR, endovascular aneurysm repair. Generic name of drugs: Cravit, levofloxacin; Brosym, cefoperazone-sulbactam; Ocillina, oxacillin; Invanz, ertapenem; Bojum, meropenem; Cexime, cefixime; Taigexyn, nemonoxacin.

A timeline describing the six episodes of K. pneumoniae infections in the 63-year-old diabetic man admitted to our hospital in 2020 is depicted in Fig. 1. In brief, the patient initially suffered left ankle necrotizing fasciitis, then left psoas muscle abscess with septic infrarenal abdominal aortic aneurysm (AAA). K. pneumoniae isolated from the left psoas muscle abscess on January 14, 2020, was collected as CSKP204002. After the first hospitalization, the patient suffered two subsequent episodes of K. pneumoniae infections. Since his third discharge on March 20, 2020, the patient was treated with oral cefixime until the fourth episode of infections which started on August 3, 2020. Later, partial hepatectomy and open cholecystectomy were performed because of the development of liver abscess with chronic granuloma and fibrosis, and three more isolates (CSKP204031, CSKP204034, and CSKP204035; Table 1) were collected from August 10 to August 27 (Table 1). The patient was hospitalized again on September 15 due to bacteremia complicated with left ankle fasciitis, bilateral lung abscess, and recurrent AAA. Aggressive debridement and drainage were applied, and four isolates (CSKP204046, CSKP204047, CSKP204048, and CSKP204061; Table 1) were collected from September 21 to October 20. After treatment with IV ceftaroline fosamil, ciprofloxacin, and tigecycline, this patient was discharged with a prescription of oral cefixime and nemonoxacin. In the final episode, K. pneumoniae CSKP204079 was collected from the patient’s abdominal drainage of the biloma at liver S4. The patient was then treated with IV taigexyn, cefixime, and meropenem until his laboratory data returned to normal on December 7, 2020.

**TABLE 1:** The antimicrobial susceptibility testing result of ST23_KL1 K. pneumoniae isolates from a single patient^*a*^

Isolate	CSKP204002	CSKP204031	CSKP204035	CSKP204034	CSKP204046	CSKP204048	CSKP204047	CSKP204061	CSKP204079
Clinical specimen for bacterial isolation	Drainage from left psoas muscle abscess	Pus from subcutaneous wound	Pus from liver abscess	Retroperitoneal ascites	Pus from subcutaneous wound	Pus from left abdominal wound	Drainage from lung abscess	Abdominal drainage	Drainage from liver biloma
Isolation time	2020.01.14 11:52	2020.08.10 09:57	2020.08.20 16:25	2020.08.27 10:14	2020.09.21 11:41	2020.09.25 09:51	2020.09.25 17:50	2020.10.20 11:04	2020.11.26 16:21
Cefoxitin	≤4	≤4	≤4	≤4	≤4	≤4	≤4	≤4	≤4
Amikacin	≤2	≤2	≤2	≤2	≤2	≤2	≤2	≤2	≤2
Gentamicin	≤1	≤1	≤1	≤1	≤1	≤1	≤1	≤1	≤1
Cefoperazone/Sulbactam	≤8	≤8	≤8	≤8	≤8	≤8	≤8	≤8	≤8
Piperacillin-Tazobactam	≤4	≤4	≤4	≤4	≤4	≤4	≤4	≤4	≤4
Ampicillin/Sulbactam	8	8	8	4	8	4	8	8	8
Ertapenem	≤0.12	≤0.12	≤0.12	≤0.12	≤0.12	≤0.12	≤0.12	≤0.12	≤0.12
Imipenem	0.5	1	≤0.25	≤0.25	1	1	2	1	1
Cefazolin	≤2	4	4	≤2	4	4	4	≤2	4
Ceftazidime	≤0.12	≤0.12	≤0.12	0.25	≤0.12	≤0.12	≤0.12	≤0.12	≤0.12
Ceftriaxone	≤0.25	≤0.25	≤0.25	≤0.25	≤0.25	≤0.25	≤0.25	≤0.25	≤0.25
Ciprofloxacin	0.5	0.5	0.5	0.5	0.5	0.5	2	0.5	0.5
Tigecycline	≤0.5	≤0.5	≤0.5	≤0.5	≤0.5	≤0.5	1	≤0.5	≤0.5

a Antimicrobial susceptibility testing was performed with standard broth micro-dilution method and interpreted based on the criteria from the Clinical and Laboratory Standards Institute (CLSI) guidelines (M100-S27).

### Comparative genomic analyses of CG23-I K. pneumoniae longitudinally isolated from recurrent infections in a single patient.

**FIG 2:**
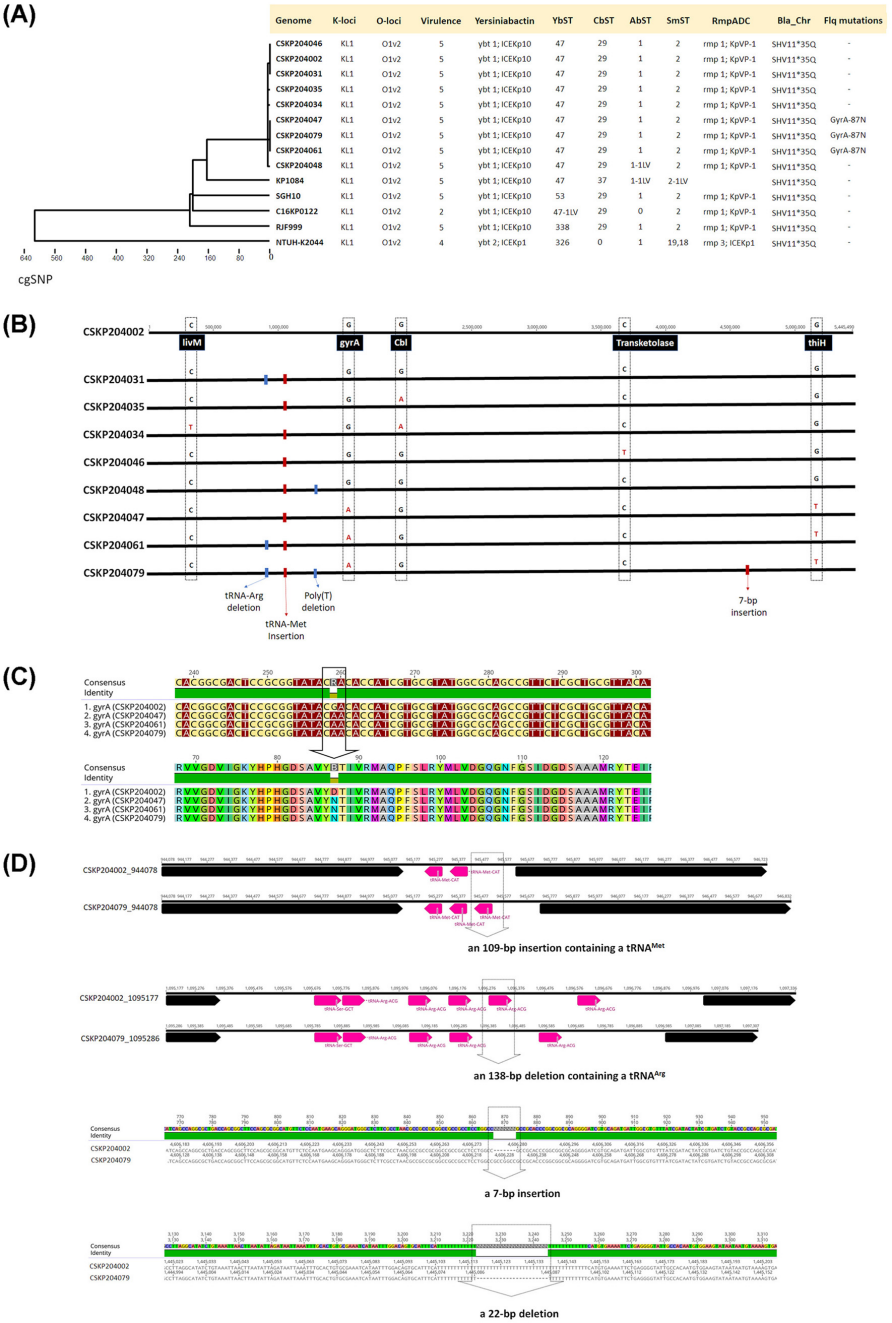
Phylogeny and comparative genomic analysis of CG23-I K. pneumoniae isolates. (A) Core genome SNP phylogeny of the nine isolates from a single patient with closely related CG23 strains, including KP1084 (NC_018522.1), SGH10 (NZ_CP025080.1), RJF999 (CP014010.1), NTHU-K2044 (AP006725.1), and C16KP0122 (CP052431.1) as the reference. Genomic features predicted in Pathogenwatch, including CPS (K-locus) genotype, LPS (O-locus) genotype, the carriage of Integrative and Conjugative Element of K. pneumoniae (ICE*KP*), sequence type of yersiniabactin (YbST), colibactin (CbST), aerobactin (AbST), salmochelin (SmST), and RmpADC, and the presence of *bla*_SHV_, and mutations related to fluoroquinolone resistance, are shown correspondingly. (B) SNP in the genome of the nine isolates. (C) Pairwise alignment of *gyrA* between CSKP204002 and CSKP204047, CSKP204061, and CSKP204079, which had a single G to A nucleotide transition, resulting in a substitution of the amino acid residue at position 87 from D to N. (D) Insertions and deletions (indels) identified in the genome of CSKP204079 compared with that of CSKP204002. Pink arrows represent tRNA genes.

To examine whether a single lineage of K. pneumoniae caused the recurrent infections of this patient, we performed Illumina MiSeq of the nine isolates and determined the complete genome sequences of CSKP204002 (the initial isolate) and CSKP204079 (the last isolate) by combining MinION long-reads with Illumina sequencing data. CSKP204002 carried a circular 5,445,490 bp chromosome and two plasmids: pLV-4002 (a 223,518 bp repB_KLEB type) and p4K-4002 (a 4,437 bp nontypeable plasmid) (Fig. S1A, C, and E). CSKP204079 harbored a 5,445,442-bp chromosome and two plasmids as those carried by CSKP204002 (Fig. S1B, D, and F). Except for a chromosome-borne *bla*_SHV11.v1^ 35Q_, no antimicrobial resistance genes were found. CSKP204002 and CSKP204079 were ST23_KL1 K. pneumoniae and phylogenetically belonged to CG23-I HvKP (12). Low nucleotide divergence across core genes of the nine genomes, with a median distance of five single nucleotide polymorphisms (SNPs) (Fig. 2A), indicated that the nine longitudinally collected isolates belonged to a single lineage originating from the initial isolate CSKP204002. Point mutations in five chromosomal genes were accumulated during the 1-year within-patient microevolution of CSKP204002. Almost all the nucleotide changes were nonsense mutations (Fig. 2B), except for the G(259) to A(259) transition leading to Asp(87) to Asn(87) substitution in GyrA (Fig. 2C) detected in the last three isolates (CSKP204047, CSKP204061, and CSKP204079). Although DNA gyrase mutations contributed to resistance to quinolones, only CSKP204079 exhibited increased MIC to ciprofloxacin from 0.5 to 2.0 μg/mL (Table 1). We aligned the chromosome of CSKP204079 against that of the initial isolate CSKP204002. Four insertions and deletions (indels) were identified, including an insertion of a 109-bp region containing a tRNA^Met^ gene, a deletion of a 138-bp region containing a tRNA^Arg^ gene, a 7-bp insertion in the region coding for a putative alanine/glycine transport protein, and a 22 bp poly-T deletion in an intergenic region (Fig. 2D).

### Genome comparison with other CG23-I K. pneumoniae.

**FIG 3:**
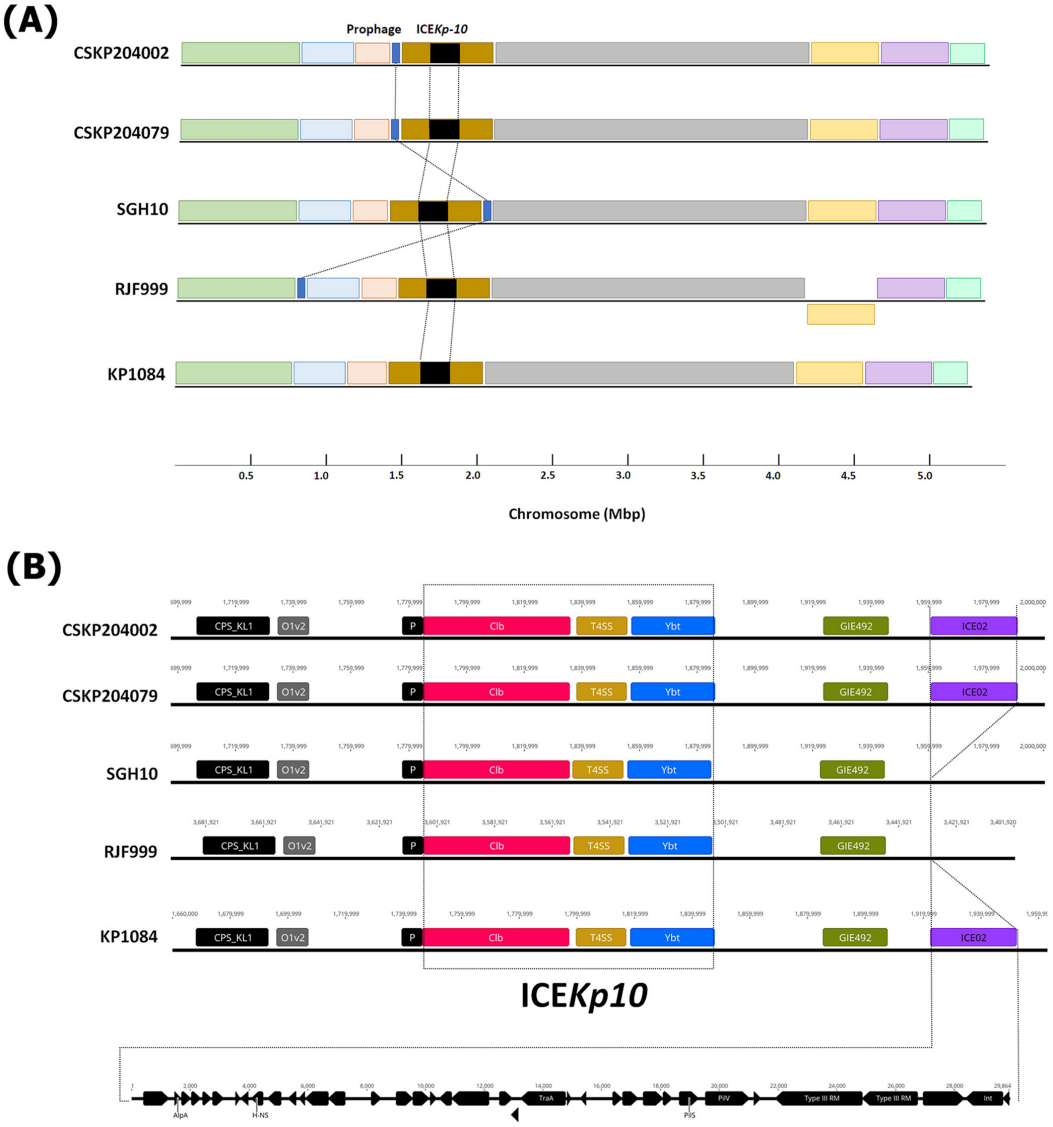
Genome comparison with closely related CG23-I K. pneumoniae. (A) Chromosomal synteny and comparison between CSKP204002, CSKP204079, and the closely related CG23-I K. pneumoniae strains, KP1084 (NC_018522.1), SGH10 (NZ_CP025080.1), and RJF999 (CP014010.1). Homologous regions with the same orientation as CSKP204002 are shown in colored modules above and inverted modules below the line. (B) Linear comparison of the ICE*Kp10* region. The genetic loci coding for the biosynthesis of colibactin (Clb), type IV secretion system (T4SS), yersiniabactin (Ybt), and microcin (GIE492) are shown in red, brown, blue, and green, respectively. Gene content of the novel ICE region (ICE02), harbored by the genomes of CSKP204002, CSKP204079, and KP1084, is shown below.

**FIG 4:**
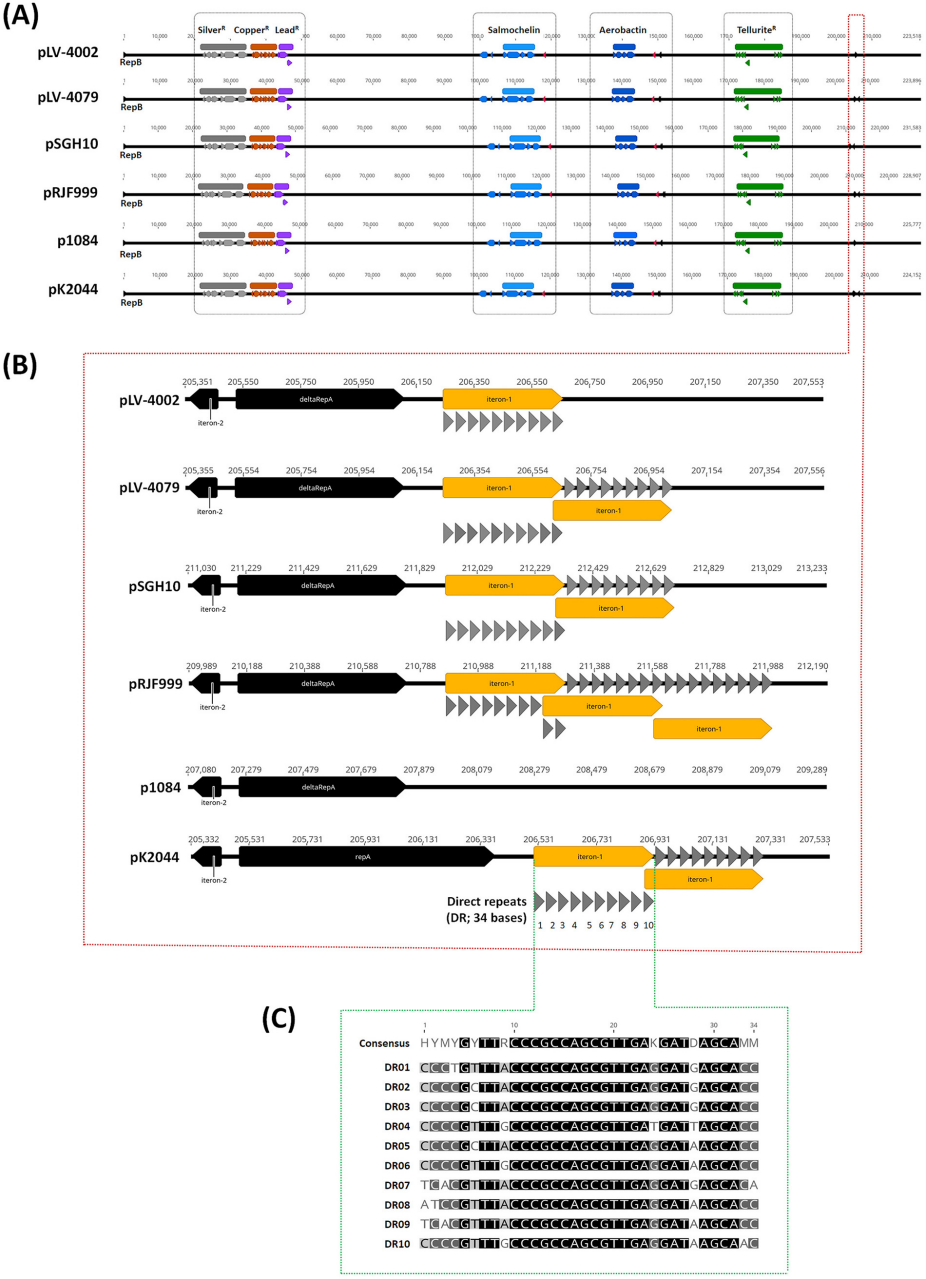
Linear comparison of the large virulence plasmids. (A) pLV-4002 (223,518 bp), pLV-4079 (223,896 bp), pSGH10 (231,583 bp), pRJF999 (228,907 bp), p1084 (225,777 bp), and pK2044 (224,152 bp), were identified in CSKP204002, CSKP204079, SGH10, RJF999, KP1084, and NTUH-K2044, respectively. The genetic loci coding for resistance to silver, copper, lead, and tellurite and the biosynthesis of salmochelin and aerobactin are shown in gray, orange, purple, green, light, and dark blue, respectively. A detailed comparison of the *repA* region is shown in (B). Adjacent to the 3′-end of the *repA* gene, the interon-1 region, 413 bp in length, was composed of 10 34-nucleotide direct repeats (DRs). Except for p1084, all the large virulence plasmids carried one to three copies of the iteron-1 region. (C) The 10 34-nucleotide direct repeats (DRs) are numbered according to their order in the iteron-1 region and are aligned by Geneious alignment (Geneious Prime 2022.1.1). Twenty-four of 34 sites are identical, and the pairwise identity reaches 89.9%.

In the clustering scheme established by Lam et al., 81 isolates were categorized within the CG23-I clade (12). The genome sequences of 4 CG23-I strains were completed. Because ED2 lacked the large virulence plasmid, we only downloaded the genome sequences of KP1084 (isolated from Taiwan in 2002; GenBank accession no. CP003785) (23), SGH10 (isolated from Singapore in 2014; GenBank accession no. CP025080) (12), and RJF999 (isolated from China at 2015; GenBank accession no. CP014010), and performed genome comparisons with CSKP204002 and CSKP204079. The chromosomal synteny and content were largely conserved among the CG23-I K. pneumoniae, except for an IS*Kpn*I-flanked 1.45-Mbp inversion in RJF999 (Fig. 3A). A novel 29.8-kb ICE region was integrated downstream of the GIE492 microcin ICE in the genomes of CSKP204002, CSKP204079, and KP1084 (Fig. 3B). All five CG23-I K. pneumoniae carried a large virulence plasmid (Fig. 4A). Within the CG23-I virulence plasmids, the genetic loci coding for resistance to silver, copper, lead, and tellurite and the biosynthesis of siderophore salmochelin and aerobactin were conserved. The CG23-I virulence plasmid was a RepB_KLEB type carrying a complete *repB* gene and a truncated form of *repA*. Whereas the virulence plasmid pK2044 (NC_006625) of K. pneumoniae NTUH-K2044 (AP006725), a ST23_KL1 reference strain outside the CG23-I sublineage, had a complete *repA* gene (Fig. 4B). Adjacent to the 3′-end of the *repA* gene, a 413-bp region consisting of 10 34-nucleotide direct repeats (DRs) was identified and named the iteron-1 region. Because of a duplication of the iteron-1 region, pLV-4079, the virulence plasmid of the last isolate CSKP204079, was 378-bp larger than pLV-4002 of the initial isolate CSKP204002 (Fig. 4B). The 10 34-nucleotide DRs of the iteron-1 region were numbered according to the order and aligned. Twenty-four of the 34 sites were identical, and the pairwise identity of DRs reached 89.9% (Fig. 4C). One to three copies of the iteron-1 region were detected on the CG23-I virulence plasmids, except for p1084 (Fig. 4B).

### Genotoxicity and virulence enhancement of the last isolate CSKP204079.

**FIG 5:**
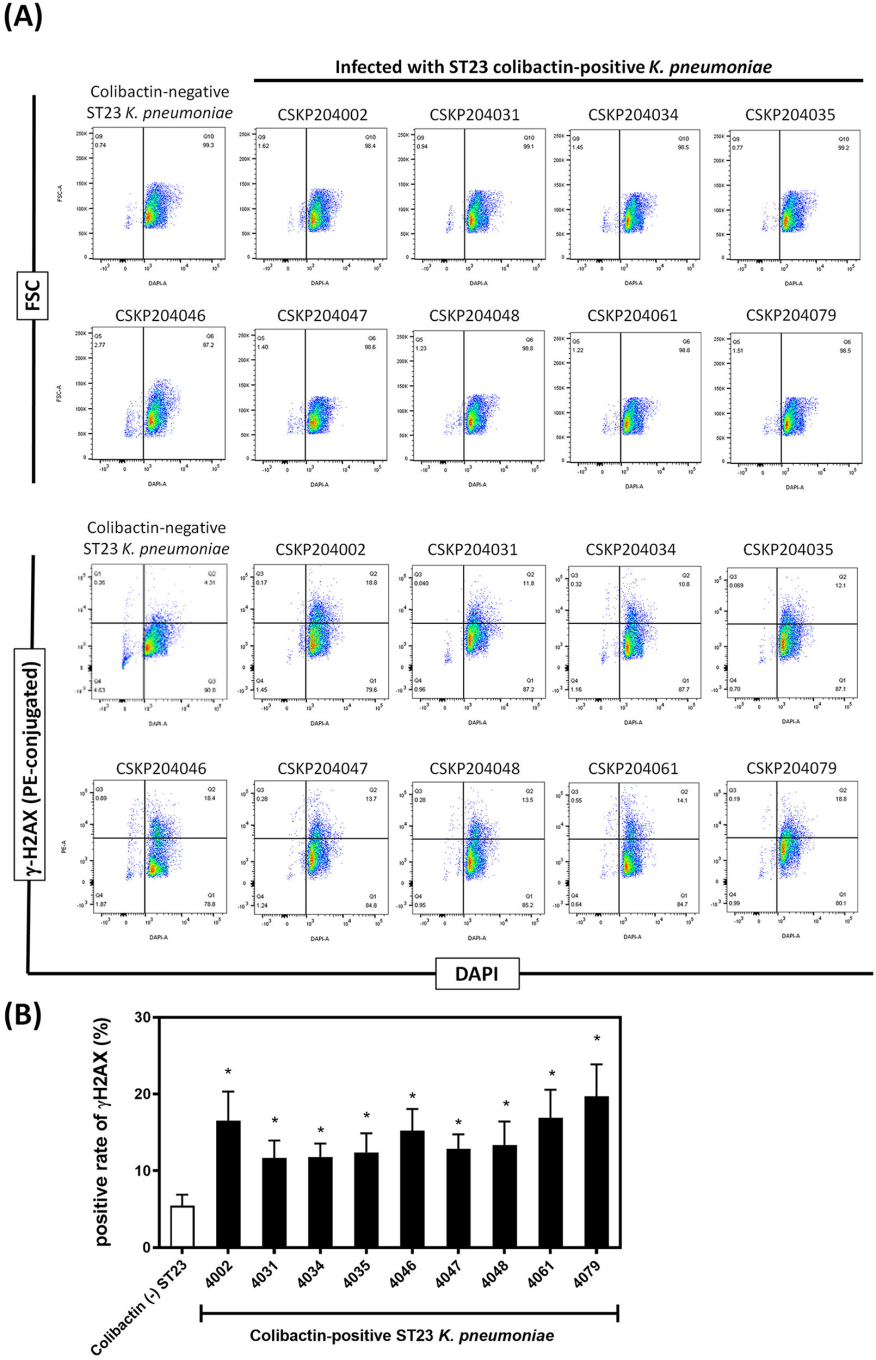
DNA damages induced by transient infections with the 9 CG23-I isolates. Confluent RAW264.7 cells were infected with individual isolates at an MOI of 100 for 1.5 h. After thorough washes, DNA damages of RAW264.7 cells were detected with PE-conjugated anti-γ-H2AX antibody. (A) Flow cytometry analysis of RAW264.7 cells infected with a colibactin-negative ST23 strain or with individual CG23-I isolates. (B) The rates of γ-H2AX^+^ RAW264.7 cells are presented as mean ± SEM from six independent experiments. One-way ANOVA determined *P* values. *, *P* < 0.05.

The genomes of all nine CG23-I isolates had a complete set of genes coding for the biosynthesis of colibactin (Fig. 3B). Thus, the production of colibactin by the nine isolates was next examined. Confluent RAW264.7 cells were transiently infected with individual CG23-I isolates with a multiplicity of infection (MOI) of 100. At 1.5-h postinfection, we examined the rates of RAW264.7 cells with DNA double-strand breaks (DSBs). By activating ATM and ATR kinases, DSBs induced phosphorylation of the histone protein H2A variant (H2AX) at serine 139 and generated γH2AX. PE-conjugated anti-γH2AX antibody was used to detect DSBs occurred in RAW264.7 cells. The rates of RAW264.7 cells with γH2AX signal were analyzed with flow cytometry (Fig. 5A). Compared with the colibactin-negative control, transient infections with any of the nine isolates significantly increased the rates of RAW264.7 cells carrying γH2AX foci (Fig. 5B).

**FIG 6:**
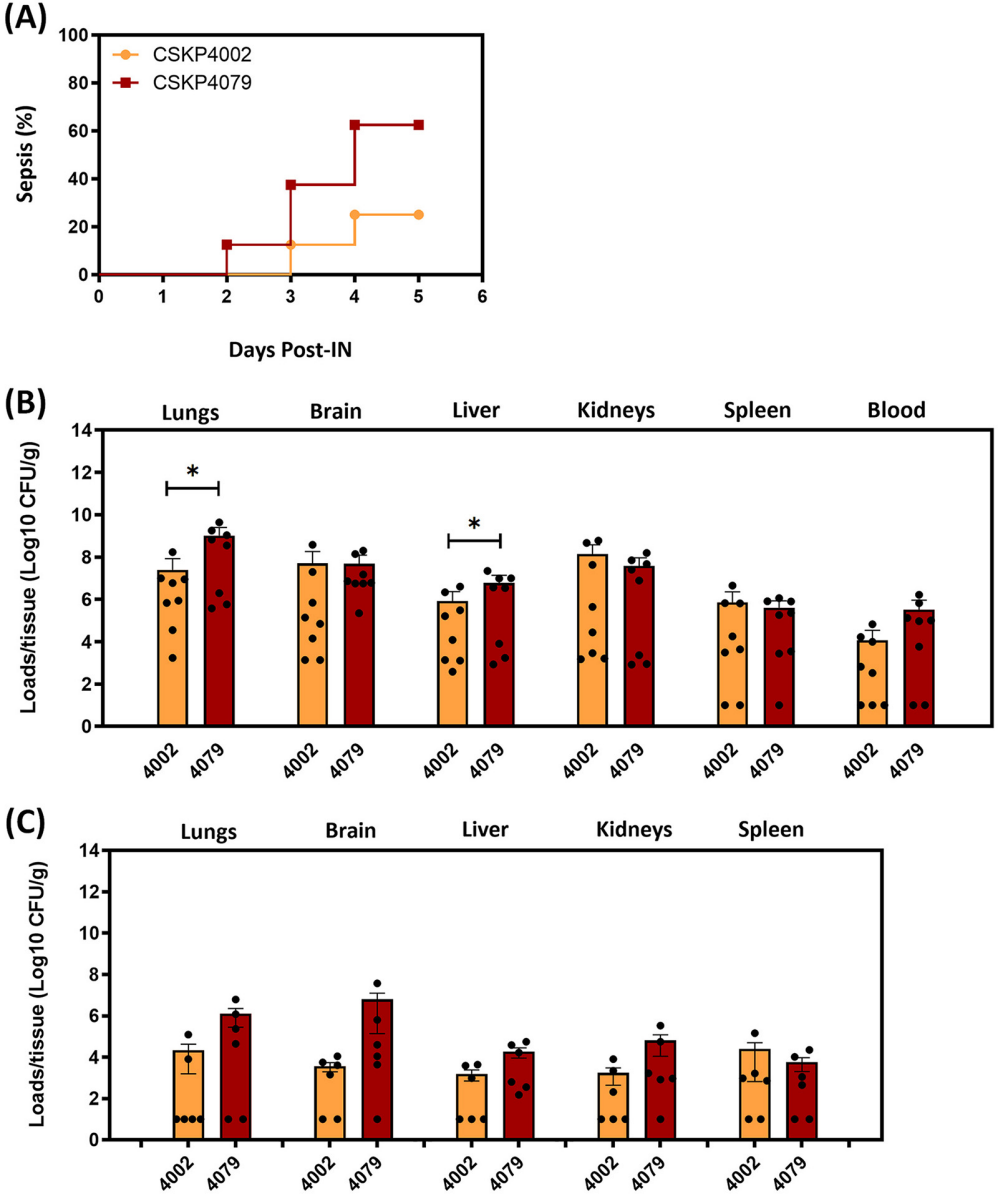
Virulence assessment of CSKP204002 and CSKP204079. (A) Groups of 8-week-old BALB/c male mice were intranasally (IN) inoculated with 1 × 10^6^ CFU of CSKP204002 or CSKP204079. The 5-day curves of sepsis developed in the mice with IN inoculation of CSKP204002 (*n *= 8; orange line) or CSKP204079 (*n *= 8; red line) were determined by Kaplan-Meier analysis using Prism 9.3.1 (GraphPad). (B) Bacterial loads in lungs, brain, liver, kidneys, spleen, and blood were determined on the day when the mice showed signs of sepsis or on the fifth day post-IN-inoculation for those with no signs of sepsis. (C) Groups of 8-week-old BALB/c male mice were orally inoculated with 5 × 10^8^ CFU of CSKP204002 or CSKP204079. Bacterial loads in the lungs, brain, liver, kidneys, and spleen were determined on the third day postoral inoculation. Dots present CFU/g of tissues from individual mice. Mean ± SEM values for the groups infected with CSKP204002 (orange) or CSKP204079 (dark red) are shown by bars using Prism 9.3.1 (GraphPad). *P* values were determined by Student's *t* test between CSKP204002 and CSKP204079. *, *P* < 0.05 (one-tailed).

Compared with the initial isolate CSKP204002, a 7-bp insertion was found at the CSKP204079 chromosome (4,606,225 to 4,606,231), which resulted from a duplication of GCCGGCC sequence and created a premature stop codon, leading to the translation of a truncated form (281 amino acids) of a putative alanine/glycine transport protein (with a full length of 485 amino acids) (Fig. 2D). To further examine whether CSKP204079 virulence was affected, we inoculated CSKP204079 and CSKP204002 into groups of 8-week-old male BALB/c mice by intranasal and oral delivery. Through an intranasal (IN) route, CSKP204079 caused sepsis in 62.5% (5/8) of BALB/c mice within 5 days, which was higher than that caused by CSKP204002 (25%; 2/8; Fig. 6A). Bacterial loads in the lungs and liver of CSKP204079-IN-infected mice were significantly increased than that of the CSKP204002-IN-infected group (Fig. 6B). On the third day postoral inoculation, CSKP204079 proliferated 58.18- and 37.65-fold more than CSKP204002 in the brain and kidney (Fig. 6C). This result suggested that the last isolate, CSKP204079, exhibited enhanced virulence than the initial isolate, CSKP204002.

**FIG 7:**
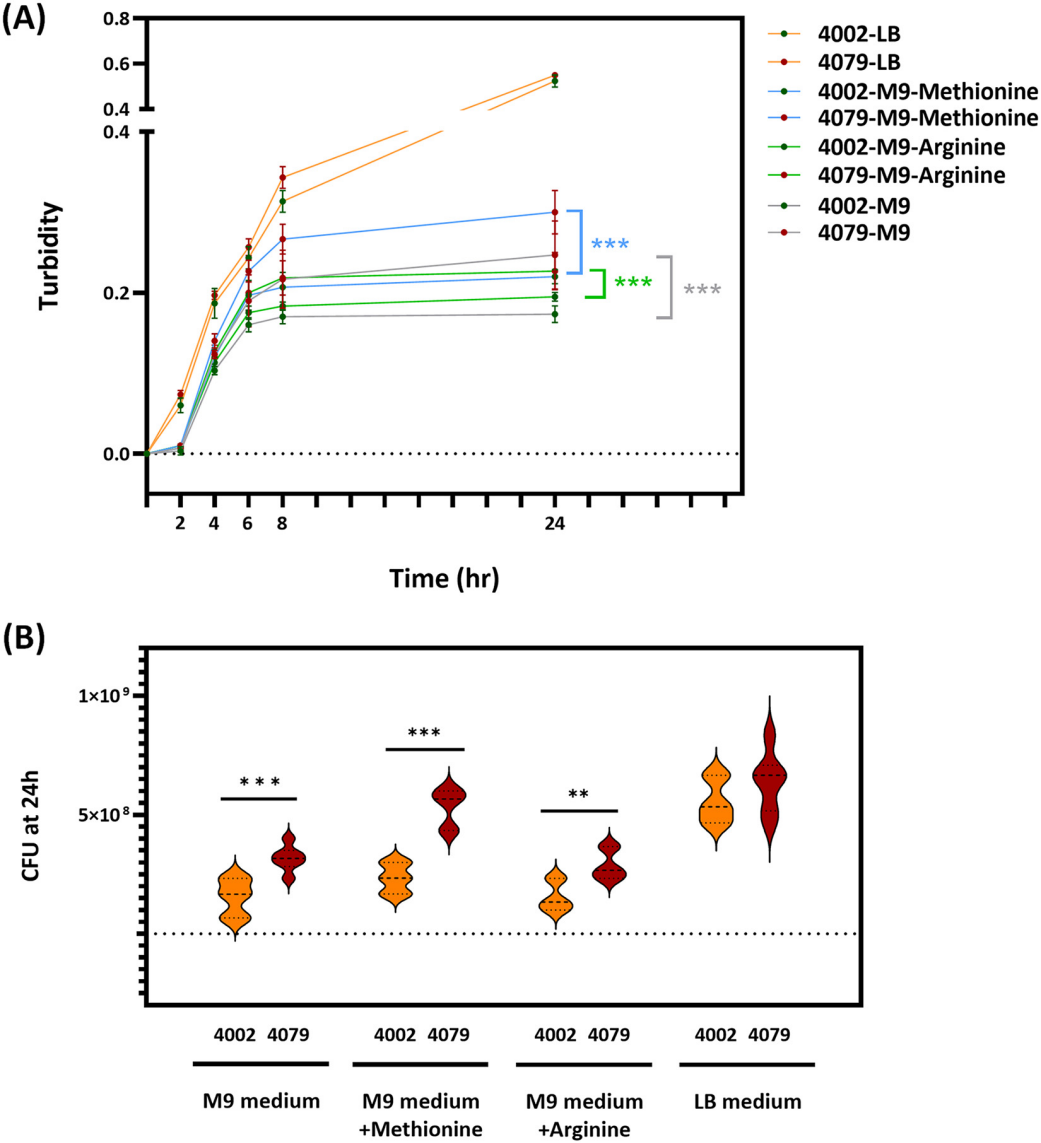
Growth assessment in M9 minimal medium. An equal amount of CSKP204002 and CSKP204079 (1 × 10^7^ CFU) was inoculated into 2 mL of M9 minimal medium supplemented with and without l-methionine (100 μg/mL) or l-arginine (100 μg/mL). (A) Growth curves were determined by turbidities at 2, 4, 6, 8, and 24 h of cultures incubated at 37°C with agitation (200 rpm). ***, *P* < 0.001 represents a significant difference between the growth curves of CSKP204002 (green dots) and CSKP204079 (red dots) from six replicates by two-way ANOVA. (B) CFU/ of cultures at 24 h were determined by a plate count method and are shown by violin plots using Prism 9.3.1 (GraphPad). *P* values were determined by Student's *t* test between CSKP204002 and CSKP204079. ***, *P* < 0.001; **, *P* < 0.01.

Compared with CSKP204002, CSKP204079 obtained an additional copy of tRNA^Met^ gene on the region between base pairs 945,305 to 945,524 but lost a copy of tRNA^Arg^ gene on base pairs 1,096,118 to 1,096,332 (Fig. 2D). The additional tRNA^Met^ in CSKP204079 was an initiator tRNA^fMet^, sharing 100% identity with the E. coli initiator tRNA^fMet^ gene (Fig. S2). The initiator-tRNA^fMet^ is crucial for initiating the protein synthesis, which positively correlates with bacterial growth rates. The fast-growing Vibrio cholerae has five initiator tRNA^fMet^ genes in their genomes (24). Because CSKP204079 carried four tRNA^fMet^ genes, one more than CSKP204002 (Fig. S2A), we examined whether the additional tRNA^fMet^ gene contributed to the growth of CSKP204079. Comparing growth curves showed that CSKP204079 grew significantly faster than CSKP204002 in the M9 minimal medium (Fig. 7A). The average 24-h yield of CSKP204079 was 2.04 times that of CSKP204002, and the difference was more pronounced by adding methionine (Fig. 7B). The growth advantage of CSKP204079 under nutrient-limited conditions could partly contribute to its virulence enhancement.

## DISCUSSION

Bacterial pathogens are constantly evolving. Beneficial traits rise from a positive selection of antibiotic treatments and immune responses during human infections. Within-patient evolution of a carbapenemase-producing ST258 K. pneumoniae has been demonstrated with genomic and phenotypic diversifications in response to antibiotic pressures (25). With high-resolution WGS techniques, we identified genomic alterations driven by within-patient selective pressures of a hypervirulent K. pneumoniae. A single CG23-I (ST23_KL1) K. pneumoniae persisted in a diabetic patient during a 1-year course, causing recurrent infections in various body sites. We collected nine isolates (CSKP204002 to CSKP204079) from the six episodes of infections during the year 2020 (Fig. 1). The maximum pairwise difference among the genomes of the nine CG23-I K. pneumoniae isolates was five SNPs (Fig. 2A), suggesting consecutive isolates of a single lineage caused the six episodes of infections. Based on the core variations among the nine genomes, the within-patient evolution rate was estimated to be 9.2 × 10^−7^ substitutions site^−1 ^year^−1^, which was 2.7 times higher than the average substitution rate of CG23 K. pneumoniae (12). Exposure to antibiotics increases the mutation rate of bacteria (26). This patient received continuous antibiotics to treat recurrent infections for 1 year (Fig. 1). It was no surprise that CG23-I K. pneumoniae adaptation was accelerated by *in vivo* challenges. Besides multiple antibiotics, the host immune defenses were also an *in vivo* stress that positively selected the variants of CG23-I K. pneumoniae with advantageous alterations in the genome.

Three isolates, CSKP204047, CSKP204061, and CSKP204079, were collected after intravenous ciprofloxacin (August 12 to August 27; September 17 to October 23) and had a GyrA-87N mutation in their genomes. Mutations in *gyrA*, frequently at the Gly-81, Ser-83, or Asp87 position, are associated with fluoroquinolone resistance in *Enterobacteriaceae* (27). However, all three isolates carrying GyrA-87N were still susceptible to ciprofloxacin (Table 1). The MIC of ciprofloxacin was increased to 2 μg/mL in CSKP204047. Besides ciprofloxacin, CSKP204047 exhibited reduced susceptibility to imipenem and tigecycline (Table 1). The addition of efflux pump inhibitor carbonyl cyanide 3-chlorophenylhydrazone (CCCP) restored the susceptibility of CSKP204047 to imipenem, tigecycline, and ciprofloxacin (Table S1). An unknown epigenomic control of efflux pumps was probably involved since no further variations were detected in the genome of CSKP204047. Apart from CSKP204002, isolated in January 2020, all the remaining isolates collected seven months later had a duplication of an initiator tRNA^fMet^ gene. Further deletion of a tRNA^Arg^ gene occurred in CSKP204048, CSKP204061, and CSKP204079 (Fig. 2B and D). Duplication or deletion of a tRNA gene was a relatively common mutation event (28). Changing the copy number of tRNA genes in the genome directly altered the composition of tRNAs and modulated protein synthesis in a bacterial cell (29, 30). With an initiator tRNA^fMet^ gene duplication, CSKP204079 grew faster than CSKP204002, particularly in a nutrient-limiting condition (Fig. 7). The within-patient-driven indel events that increase the copy number of initiator tRNA^fMet^ probably conferred CG23-I K. pneumoniae an adaptive advantage by boosting the growth rate inside the host.

Clonal group CG23, consisting of sequence types ST23, ST26, ST57, and ST163, is one of the significant clones accounting for HvKP (31). Approximately 80% of CG23 HvKP could be further grouped into CG23-I, a sublineage estimated to emerge ~100 years ago (12). The nine longitudinally collected isolates, CSKP204002 to CSKP204079, belonged to CG23-I and had restricted genomic divergence with the reference CG23-I strains, KP1084, SGH10, and RJF999 (Fig. 3). One of the genomic features distinguishing CG23-I from other CG23 sublineages was the carriage of the colibactin locus by ICE*Kp10* (17). The production of colibactin that caused DNA damage to RAW264.7 cells was detected in all nine isolates (Fig. 5). We used a flow-cytometry method to quantify the ratio of cells with DNA damage by detecting γ-H2AX foci, a marker of DNA double-strand breaks. Compared with the colibactin-negative control (5.46 ± 1.43), individual CG23-I isolates significantly increased the γ-H2AX^+^ rates of cells after a 1.5-h transient exposure, ranging from 11.65 ± 2.29 (CSKP204031) to 19.69 ± 4.18 (CSKP204079) (Fig. 5). In addition to genotoxicity, colibactin was also a virulence determinant (19, 22). With the carriage of colibactin locus, CG23-I K. pneumoniae is considered a group of HvKP exhibiting higher virulence than other lineages. Colibactin was positively correlated with HvKP colonization and increased the likelihood of causing severe complications in BALB/c mice, such as meningitis (22). Like most CG23-I HvKP, all nine isolates collected from this patient were susceptible to the antibiotics (except ampicillin; Table 1). Nevertheless, we hardly eradicated the infection foci of this lineage of K. pneumoniae despite aggressive treatments (Fig. 1). This patient had type 2 diabetes with unstably controlled levels of blood sugar. Although hyperglycemia-related impairment of immune response provided an immune-tolerant niche for K. pneumoniae (32), the carriage of CG23-I-specific virulence factors, such as colibactin, might also contribute to the persistence of this challenging pathogen.

Human infections can be viewed as natural *in vivo* selection for bacterial pathogens. Unlike Pseudomonas aeruginosa adaptation to the lungs of cystic fibrosis individuals with gain and loss of genes (33), the genome of CG23-I K. pneumoniae was relatively stable during the 1-year course of infections. Besides tRNA^Met^ duplication and tRNA^Arg^ deletion, only one coding indel occurred. The 7-bp duplication of GCCGGCC, creating a premature stop codon that disrupted the translation of a putative alanine/glycine transport protein, was detected in the last isolate CSKP204079 (chromosomal position: 4,606,225 to 4,606,231) (Fig. 2D). Even with a truncation of this transporter, CSKP204079 virulence was not weakened but enhanced in the BALB/c model compared with the initial isolate CSKP204002 (Fig. 6). Genomic alterations that were positively selected through the 1-year within-patient pathoadaptation probably contributed to the virulence enhancement of CSKP204079. Besides increasing the copy number of an initiator tRNA^fMet^ (Fig. 2D; Fig. S2), CSKP204079 also had its large virulence plasmid evolve with duplication of an iteron region. Like pSGH10 and pRJF999, the large virulence plasmids carried by the nine isolates had a truncated form of the *repA* gene (Fig. S3A) flanked by two iteron regions (Fig. 4B). Iterons are directly repeated DNA sequences that regulate the copy number of stringent plasmids (34). The formation of plasmid dimer blocks replication by binding Rep proteins with iteron sequences that handcuff them at the replication origin (35). Two iteron regions were identified on the large virulence plasmid, the 102-bp iteron-2, and the 413-bp iteron-1 region. By overlapping with the replication origin (36), the conserved iteron-2 region was a primary cis-element controlling initiation of plasmid replication. The iteron-1 region was outside the initiation site and had variable copies on different plasmids (Fig. 4B). The large virulence plasmid pLV-4079 of CSKP204079 had two copies of the iteron-1 region. In contrast, the plasmids carried by the other isolates were identical to pLV-4002 with only one copy of the iteron-1 region (Fig. S3B). The copy number of iterons outside the initiation site has been demonstrated to be negatively correlated with the copy number of plasmids (37). Under *in vitro* LB growth, the copy number of pLV-4079 (two copies of iteron-1) was 1.33 ± 0.07, less than that of pLV-4002 (one copy of iteron-1; copy number: 2.43 ± 0.23) and p1084 (no iteron-1; copy number: 3.00 ± 0.68) (Table S2). However, the replication of pLV-4079 was increased to 2.03 ± 0.21 upon K. pneumoniae CSKP204079 exposure to HCT116 cells. In contrast, the copy number of pLV-4002 and p1084 was reduced to 2.25 ± 0.12 and 1.80 ± 0.07, respectively, in the coculture with HCT116 cells. Duplication of the iteron-1 region might contribute to more flexible control of replication of the large virulence plasmid pLV-4079 to help CSKP204079 maintain a balance between rapid growth (less plasmids) and hypervirulence (more plasmids) in response to different environmental signals. Further studies are needed to fully discover the underlying mechanism.

Collectively, we characterized the within-patient microevolution of CG23-I K. pneumoniae. The genome of CG23-I K. pneumoniae remained relatively stable throughout the infectious course, as revealed by the limited genomic divergence among the nine longitudinally collected isolates. Selective pressures from continuous use of antibiotics favored point mutations contributing to bacterial resistance to antibiotics. The duplication of an initiator tRNA^fMet^ gene helped CG23-I K. pneumoniae proliferate to reach a maximal population size during infections. For longer persistence inside a human host, the large virulence plasmid evolved with more flexible control of replication through duplication of the iteron-1 region. Virulence enhancement of the last isolate CSKP204079 suggested a positive correlation between the genomic alterations and within-patient pathoadaptation of CG23-I K. pneumoniae.

## MATERIALS AND METHODS

### K. pneumoniae isolates.

A 63-year-old diabetic man admitted to Chung Shan Medical University Hospital (CSMUH) suffered from K. pneumoniae infections (Fig. 1). An isolate was collected from each K. pneumoniae-positive specimen and stored at −80°C for subsequent analysis. Species identification was performed with the Bruker MALDI Biotyper, and antimicrobial susceptibility testing was performed with the Phoenix Automatic Microbiology System (BD Diagnostics, MD, USA) and CLSI breakpoints (M100-S27). The CSMUH Institute Review Board approved this study (IRB CS19108). All methods were performed following relevant guidelines and regulations.

### Whole-genome sequencing and bioinformatics analysis.

From six episodes of infections (Fig. 1), nine isolates, CSKP-204002, -204031, -204034, -204035, -204046, -204047, -204048, -204061, and CSKP-204079, were collected from various specimens, including psoas muscle abscess, subcutaneous wound, retroperitoneal ascites, liver abscess, abdominal drainage, lung abscess, and liver biloma (Table 1), and were subjected to WGS by a Illumina MiSeq sequencer (Illumina, San Diego, USA) with the method described previously (38). Moreover, CSKP204002 (the initial isolate) and CSKP204079 (the last isolate) were sequenced by Nanopore long-read sequencing technique with MinION sequencer according to the standard protocol provided by the manufacturer (Oxford Nanopore Technologies, Oxford, UK). Hybrid assemblies of Illumina short-reads and MinION long-reads were yielded from Unicycler *v.*0.4.8 (39). Assemblies with a size ≤ 1,000 kb containing plasmid replicons were extracted from the assembly graph with BANDAGE *v.*0.8.1 (40). Whole-genome profiling, including virulence-associated loci and antimicrobial resistance genes, was performed by uploading the contigs generated from *de novo* assemblies of Illumina sequence data onto Pathogenwatch (https://pathogen.watch/) (41). Single-nucleotide polymorphisms (SNPs) calling was performed by mapping the Illumina reads of the 9 isolates and the genomes of other CG23 K. pneumoniae, including KP1084 (NC_018522.1), SGH10 (NZ_CP025080.1), RJF999 (CP014010.1), and NTHU-K2044 (AP006725.1) onto the complete sequence of C16KP0122 (CP052431.1) as the reference. Core genome SNP phylogeny was generated with the Single linkage method using BioNumerics *v.*7.6.3 (Applied Maths, Belgium). Comparative sequence alignments were performed with Geneious Prime 2022.1.1 (Biomatters, New Zealand).

### Examination of DNA damages by flow cytometry-based detection of γ-H2AX.

RAW264.7 murine macrophage (BCRC #60001, Taiwan) cells were maintained in 5% CO_2_ at 37°C in Dulbecco’s modified Eagle’s Medium with 10% fetal bovine serum. After seeding onto 6-well culture plates at a density of 2 × 10^6^ cells/well, RAW264.7 cells were synchronized in serum-free medium for 16 h and then transiently infected with CSKP204002, CSKP204031, CSKP204034, CSKP204035, CSKP204046, CSKP204047, CSKP204048, CSKP204061, and CSKP204079 at MOI of 100. After 1.5-h transient infection, RAW264.7 cells were treated with the Foxp3 Transcription Factor Staining Buffer Set (eBioscience 00-5523-00) by the manufacturer's instruction and hybridized with 1 μg of PE-conjugated anti-γ-H2AX (Ser139) monoclonal antibody (eBioscience 12-9865-42). After incubation at 4°C for 1 h, cells were washed and analyzed with a BD FACS Canto II cytometer (Becton Dickinson). Flow cytometry data were analyzed with FlowJO *v*10.7 (Becton Dickinson), and the rates of γ-H2AX-positive cells were quantified as previously described (42).

### Virulence assessment.

We obtained a total of nine isolates from different infectious foci of a single patient in 1 year. With low nucleotide divergence (a median distance of five SNPs), these isolates were considered consecutive variants of a single ancestor. CSKP204002 was the first isolate collected from the left psoas muscle abscess. Subsequent relapse of infections in multiple body sites of this patient suggested that the variants of CSKP204002 were capable of disseminating systematically. CSKP204079 was the isolate collected from abdominal drainage in the final episode of infections. We used the BALB/c model, which we established previously for examining the virulence of CG23-I K. pneumoniae strain 1084 (21, 22), to compare the virulence of CSKP204002 and CSKP204079 in terms of their ability to cause systemic infections. Male BALB/c mice were purchased from BioLasco (Taiwan Co., Ltd.) at 7-week-old, and allowed to acclimatize in the animal house for 1 week. Bacterial inoculation was carried out by oral and intranasal delivery. (i) Oral inoculation: Groups of mice received streptomycin (500 μg/mL) in drinking water for three consecutive days and then orally inoculated with 5 × 10^8^ CFU of CSKP204002 and CSKP204079, respectively. All the mice survived the observation period and were sacrificed on the third day postinoculation. The liver, spleen, kidneys, lungs, and brain were retrieved for bacterial enumeration. (ii) Intranasal inoculation: groups of mice were intranasally inoculated with 1 × 10^6^ CFU of CSKP204002 and CSKP204079, respectively. On the day when individual mice exhibited signs of sepsis, including ruffled fur with shivering and piloerection, severe dyspnea with no relocation, hunched posture with mostly closed eyelids, and a reduction in body temperature >5°C (43), humane endpoints were applied to minimize the suffering of mice. The survived mice were sacrificed on the fifth day postinoculation. The liver, spleen, kidneys, lungs, brain, and blood were retrieved for bacterial enumeration. The experiments were performed following the Guide for the Care and Use of Laboratory Animals of the National Research (44). The protocols were approved by the Institutional Animal Care and Use Committee of Chung Shan Medical University (Permit number: 2489).

### Growth assessment in M9 minimal medium.

To examine whether the duplication of the initiator tRNA^fMet^ gene contributed to the growth of CSKP204079, we comparatively measured the growth curve of CSKP204002 (three initiator tRNA^Met^ genes) and CSKP204079 (four initiator tRNA^Met^ genes) in M9 minimal medium (M9 minimal salts, 0.02% glucose, 2 μM MgSO_4_, 0.1 μM CaCl_2_) supplemented with and without l-methionine (100 μg/mL) or l-arginine (100 μg/mL). Turbidity of bacterial cultures was detected with a Microscan Turbidity Meter (Beckman coulter) at indicated time points. The number of CFU was measured by a conventional plate count method.

### Data availability.

WGSs determined in this study are publicly available in GenBank under accession no. PRJNA818087. All data generated or analyzed during this study are included in this article. Any additional information will be made available from the corresponding author upon reasonable request.
