# Supplementary material for: Microevolution of CG23-I Hypervirulent Klebsiella pneumoniae during Recurrent Infections in a Single Patient
Source: Microbiol Spectr. 2022 Sep 21;10(5):e02077-22. doi: 10.1128/spectrum.02077-22 (PMC9602619; doi:10.1128/spectrum.02077-22)
Supplement: Supplemental file 1 — Tables S1 and S2 and Fig. S1 to S3. Download spectrum.02077-22-s0001.pdf, PDF file, 4.0 MB [file spectrum.02077-22-s0001.pdf]

Supplemental Table 1. The antimicrobial susceptibility testing of CSKP204047.

	**MIC (μg/ml)**[a]	
		+CCCP[b]
Imipenem	2	0.5
Ciprofloxacin	2	0.5
Tigecycline	1	≤0.5

[a]Antimicrobial susceptibility testing was performed with a standard broth micro-dilution method.

[b]Carbonyl cyanide 3-chlorophenylhydrazone (CCCP; C2759 Sigma-Aldrich) was added into the wells with a final concentration of 25 μg/ml.

Supplemental Table 2. Estimated plasmid copy number (PCN) of the large virulence plasmid in CG23-I *K. pneumoniae* strain CSKP204002, CSKP204079, and KP1084.

	***In vitro* LB growth**[a]			**Coculture with HCT116 cells**[a]		
	ΔCt[b]	PCN ($2^{-\Delta Ct}$)[c]	CV (%)[d]	ΔCt[b]	PCN ($2^{-\Delta Ct}$)[c]	CV (%)[d]
**CSKP204002**	-1.31±0.13	**2.43±0.23**	1.38±0.67	-1.02±0.15	**2.03±0.21**	0.73±0.63
**CSKP204079**	-0.41±0.07	**1.33±0.07**	1.26±0.45	-1.16±0.08	**2.25±0.12**	1.11±0.64
**KP1084**	-1.55±0.34	**3.00±0.68**	1.28±0.75	-0.85±0.05	**1.80±0.07**	1.16±0.81

[a]Total genomic DNA was extracted from *K. pneumoniae* CSKP204002, CSKP204079, and KP1084, which were grown in LB medium or were cocultured with HCT116 cells in RMPI1640 medium at 37°C for 3 hours by using Qiagen DNeasy Blood & Tissue Kit.
[b]Real-time qPCR amplification was performed using CFX384 Real-Time PCR Detection System (Bio-Rad). The threshold cycle (Ct) was analyzed with CFX Maestro Software. Two primer sets specific to the *silA* gene (forward-GTT GCG GCC TTG TCC ATT TT-3', reverse-AGG TTT GAG CTG GAT CGT GG-3'), which is a single-copy gene of the large virulence plasmid, and to the *rpoS* gene (forward-GTA CTT GAC GCC ACT CAG CT-3', reverse-GTC GAA AAA CGG AAC CCA CG-3'), which is a single copy gene of *K. pneumoniae* chromosomal DNA, were used. $\Delta Ct=[\text{adjusted Ct value}]_{silA}-[\text{adjusted Ct value}]_{rpoS}$
[c]Plasmid copy number (PCN) of the large virulence plasmid was estimated by relative quantification of Ct values, showing the amount of *silA* (on the large virulence plasmid) relative to the amount of *rpoS* (on chromosome) in *K. pneumoniae* CSKP204002, CSKP204079, and KP1084 grown in LB medium or coculture with HCT116 cells. Five serial dilutions of genomic DNA corresponding to 1×10^7 CFU/μl to 1×10^4 CFU/μl were used to determine a standard curve. Ct values in each dilution were measured in triplicates. PCR amplification efficiency was calculated as $E=10^{-1/slope}-1$, ranging from 75.8% to 99.3%. $E_{100\%}$ adjusted $Ct=Log_2(1+E)^{Ct}$. Adjusted Ct values of *silA* and *rpoS* from the five dilutions of individual genomic DNA were used to calculate ΔCt, presented as mean ± standard deviation. The $2^{-\Delta Ct}$ calculation determined the plasmid copy number (PCN).
[d]CV (%): coefficient of variation values (%) are presented as mean ± standard deviation.

(A)

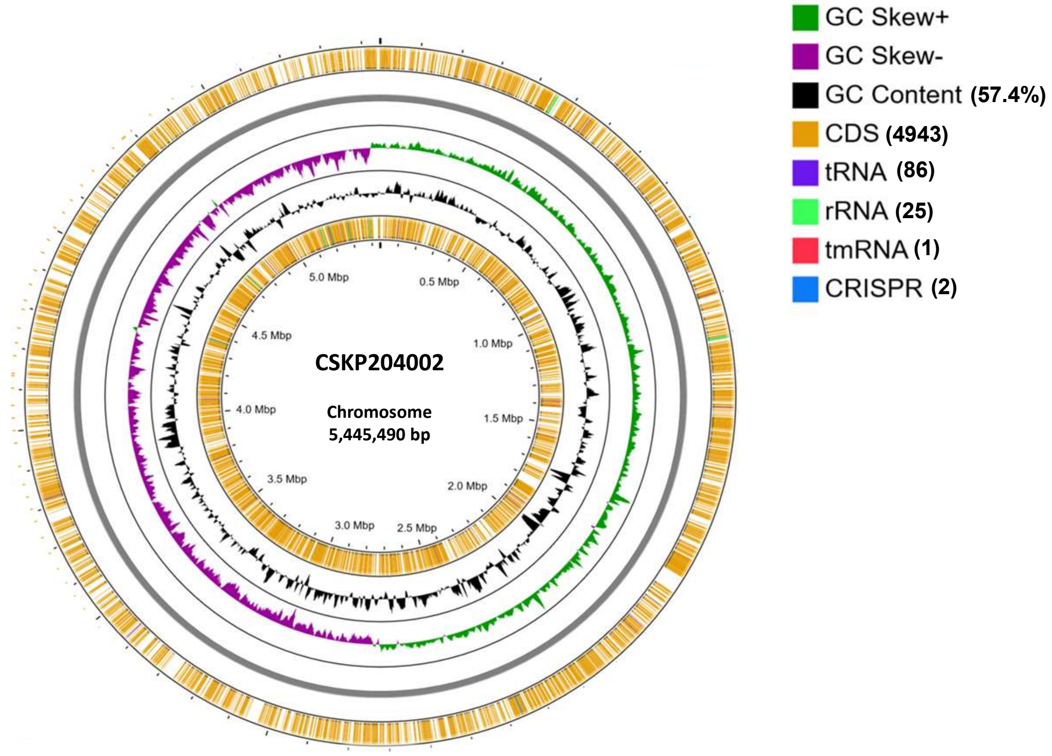

(B)

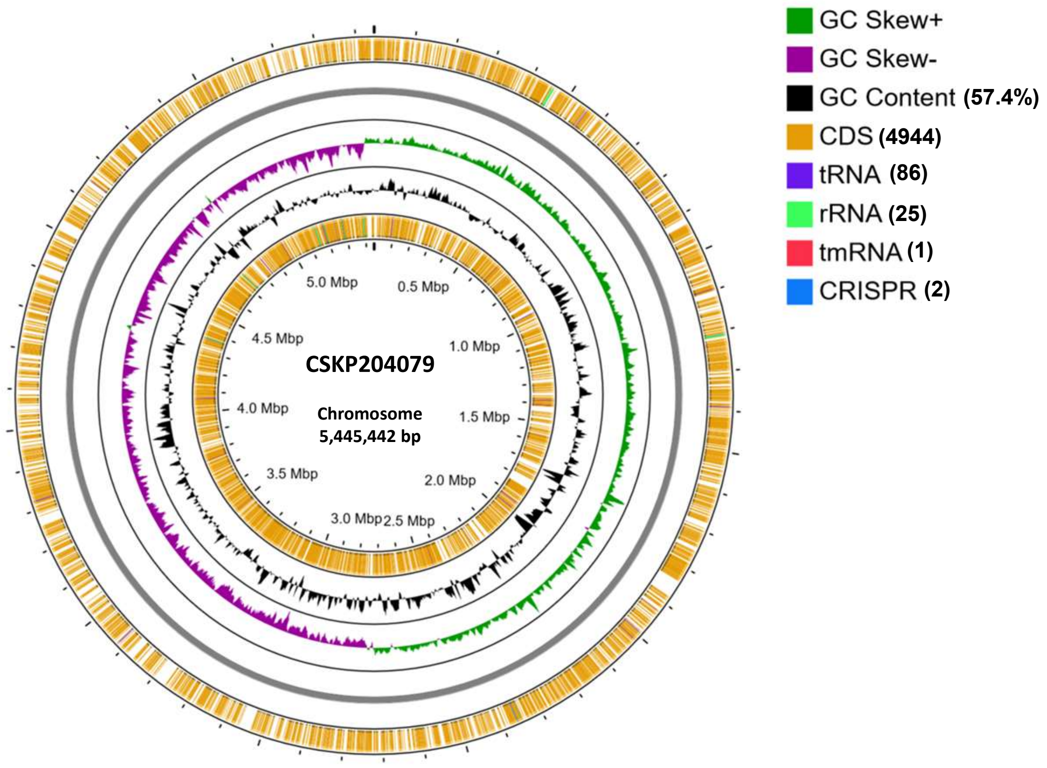

Supplemental Figure 1 (A and B). Genome map of chromosome DNA from CSKP204002 (A) and CSKP204079 (B), generated by CGView (https://cgview.ca/). Circles from outside to inside showing: coding regions (CDS) predicted on forward (outer circle) and reverse strands (inner circle), regions above (green) and below (purple) the average GC skew, and GC content (black).

(C)

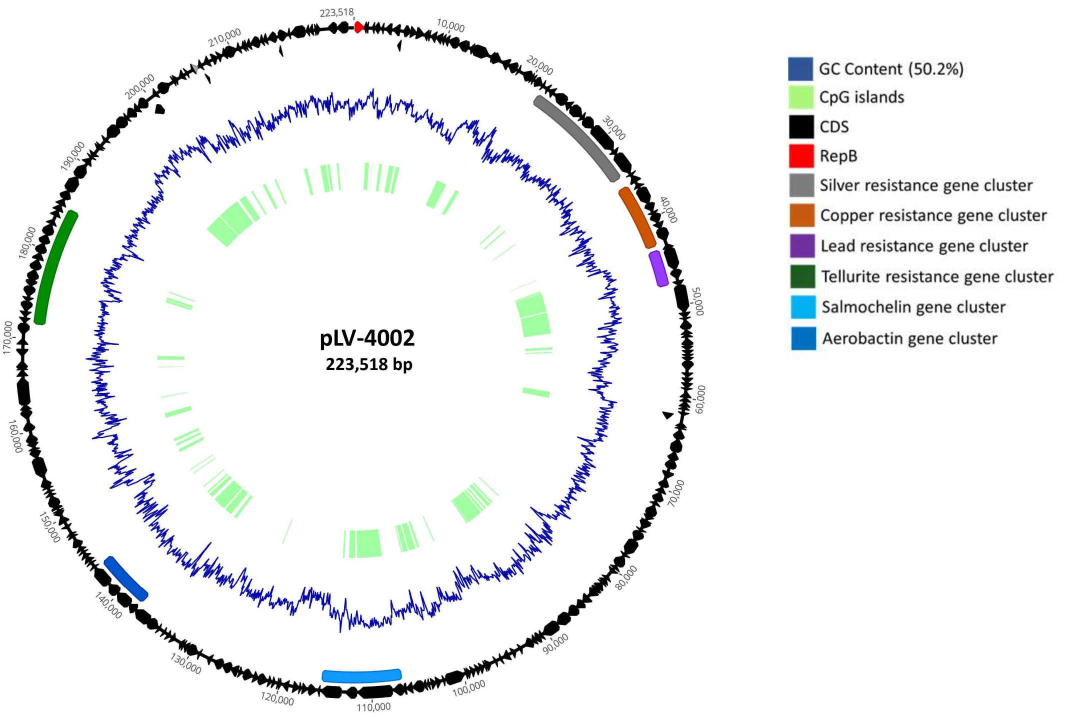

(D)

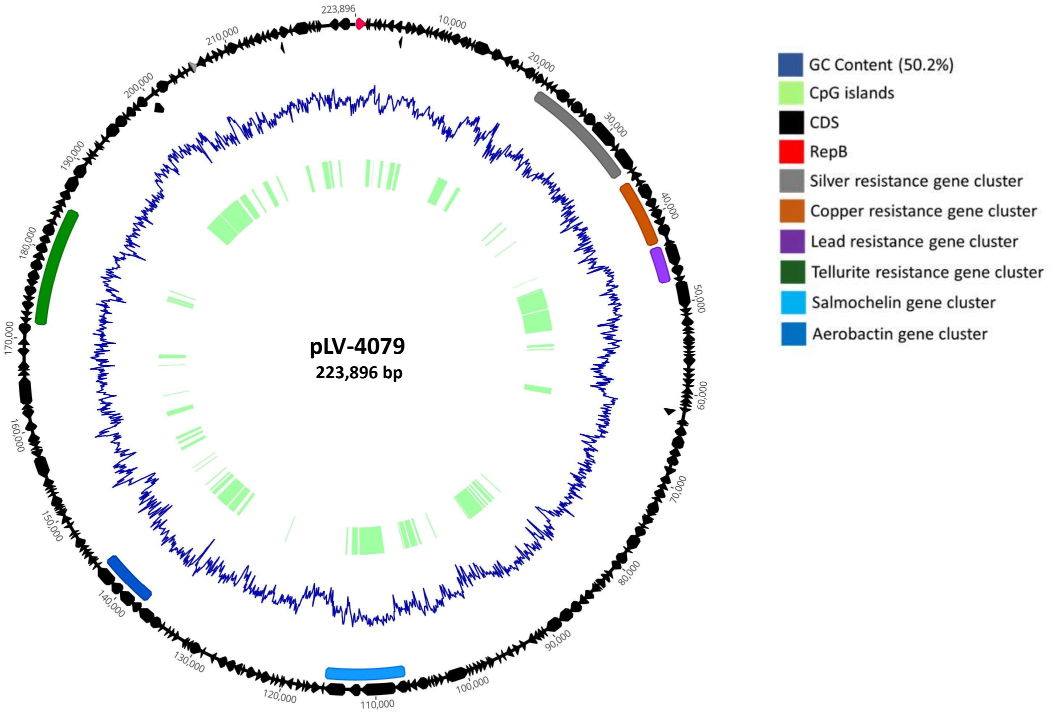

Supplemental Figure 1 (C and D). Genome map of the large virulence plasmid in CSKP204002, named pLV-4002 **(C)**, and CSKP204079, named pLV-4079 **(D)**, generated by Geneious Prime® 2022.1.1. Circles from outside to inside showing: coding regions (CDS; black), gene clusters coding for resistance to silver (grey), copper (brown), lead (purple), tellurite (dark green) and biosynthesis of salmochelin (light blue) and aerobactin (ocean blue), the GC content (dark blue), and the predicted CpG islands (light green).

(E)

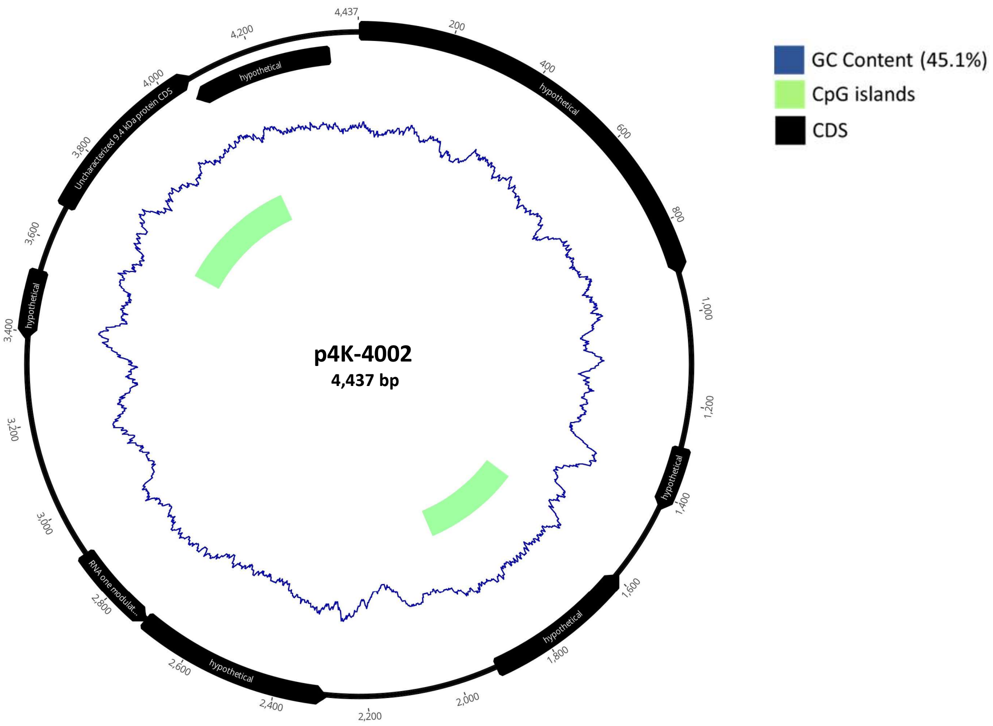

(F)

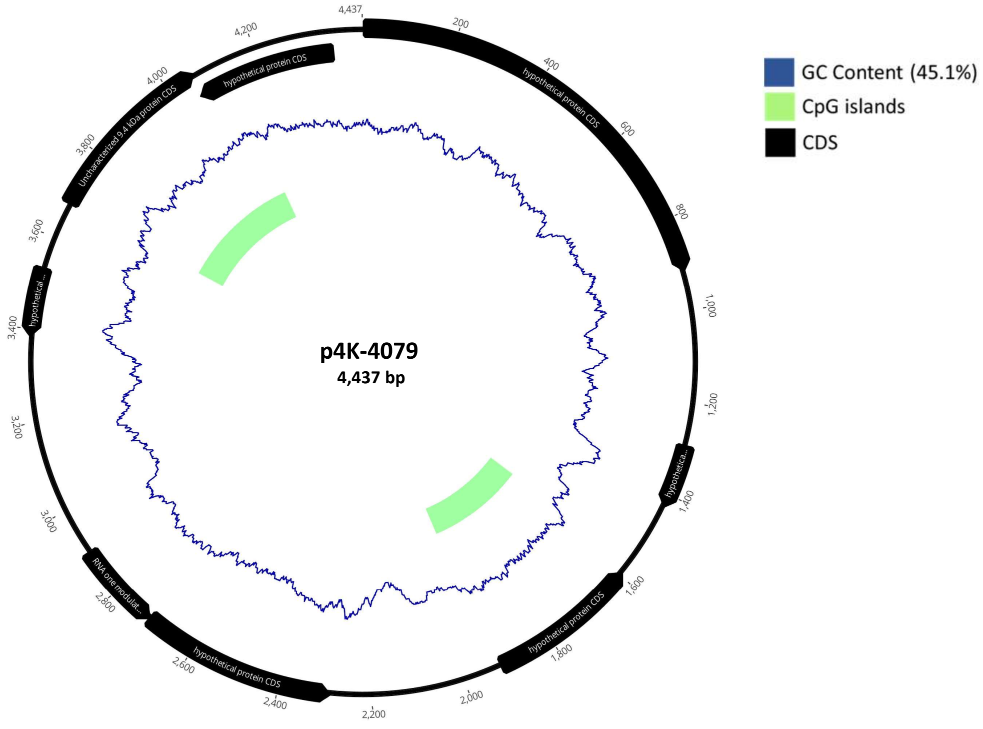

Supplemental Figure 1 (E and F). Genome map of the 4-Kb plasmid in CSKP204002, named p4K-4002 **(E)**, and CSKP204079, named p4K-4079 **(F)**, generated by Geneious Prime® 2022.1.1. Circles from outside to inside showing: coding regions (CDS; black), the GC content (dark blue), and the predicted CpG islands (light green).

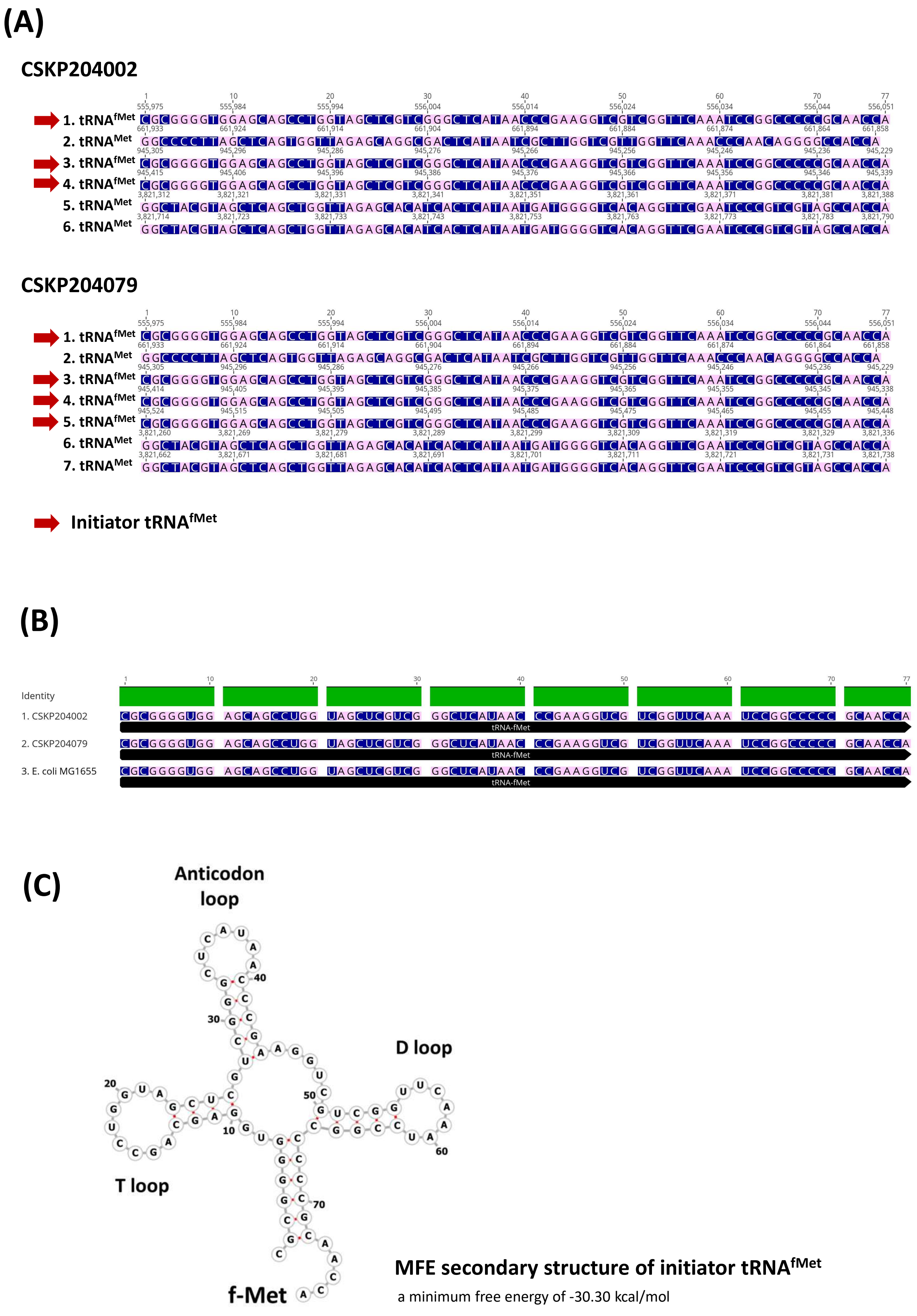

Supplemental Figure 2. Initiator tRNA^{fMet} in CSKP204002 and CSKP204079. **(A)** Genes encoding initiator tRNA^{fMet} are indicated with red arrows in CSKP204002 and CSKP204079. **(B)** Alignment of the initiator tRNA^{fMet} sequences carried by CSKP204002, CSKP204079, and *Escherichia coli* MG1655. **(C)** MFE secondary structure of initiator tRNA^{fMet} in CSKP strains predicted by RNAfold WebServer (http://rna.tbi.univie.ac.at/).

(A)

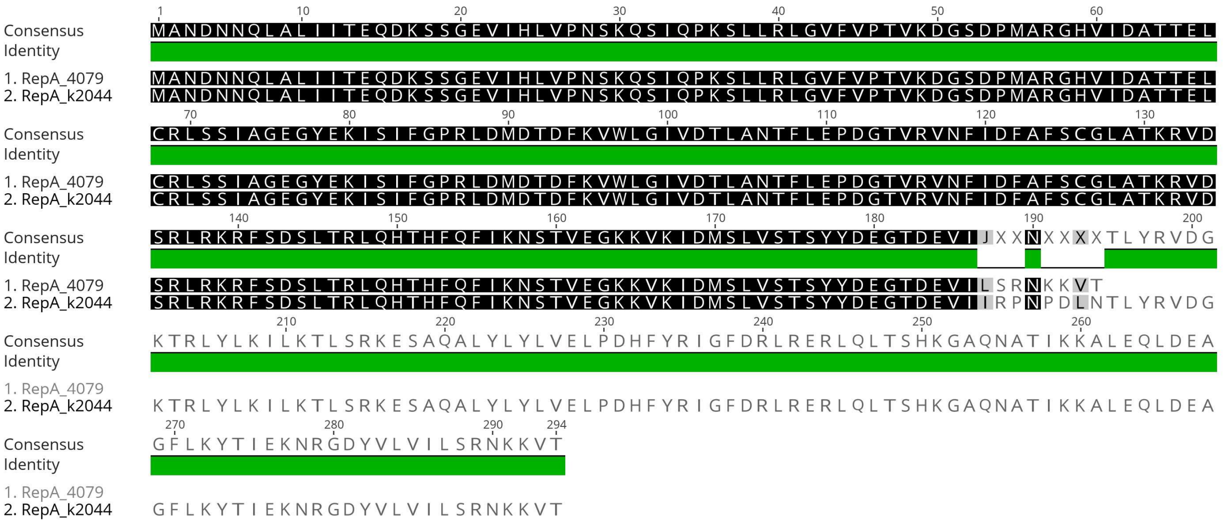

(B)

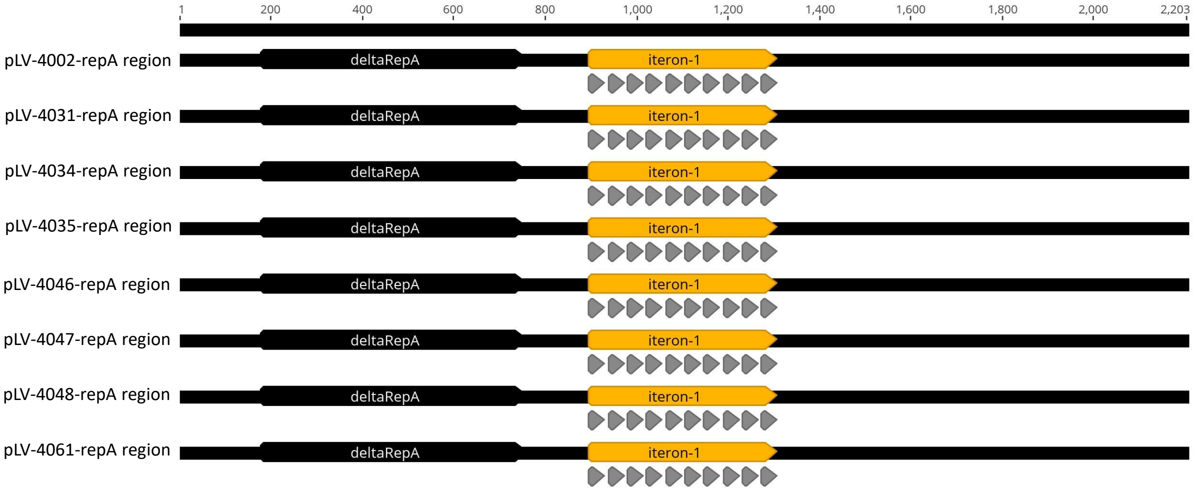

Supplemental Figure 3. (A) Alignment of RepA proteins encoded by a complete and truncated form of *repA* gene carried by pK2044 and pCS204079, respectively. **(B)** Pairwise alignment of the *repA* region with iteron-1 on the large virulence plasmid carried by the 8 CG23-I *K. pneumoniae* isolates.
